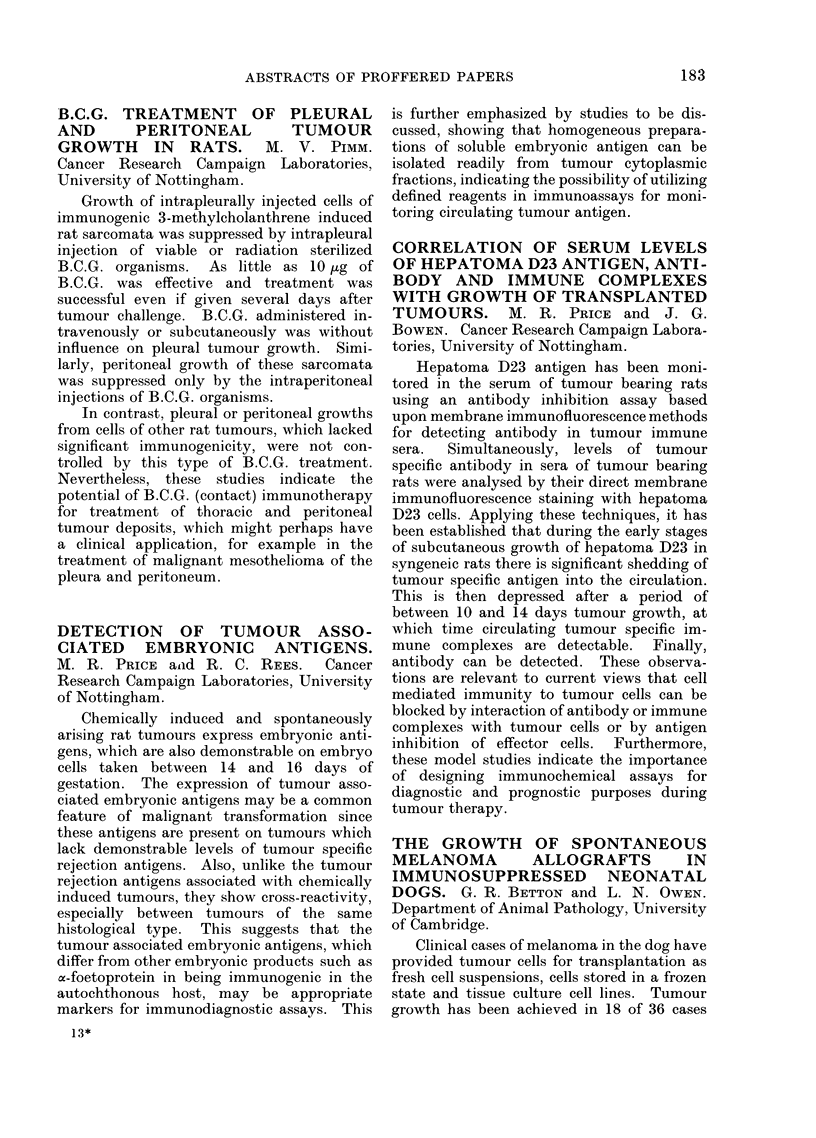# Proceedings: Correlation of serum levels of hepatoma D23 antigen, antibody and immune complexes with growth of transplanted tumours.

**DOI:** 10.1038/bjc.1974.167

**Published:** 1974-08

**Authors:** M. R. Price, J. G. Bowen


**CORRELATION OF SERUM LEVELS OF HEPATOMA D23 ANTIGEN, ANTIBODY AND IMMUNE COMPLEXES WITH GROWTH OF TRANSPLANTED TUMOURS.** M. R. Price and J. G. Bowen. Cancer Research Campaign Laboratories, University of Nottingham.

Hepatoma D23 antigen has been monitored in the serum of tumour bearing rats using an antibody inhibition assay based upon membrane immunofluorescence methods for detecting antibody in tumour immune sera. Simultaneously, levels of tumour specific antibody in sera of tumour bearing rats were analysed by their direct membrane immunofluorescence staining with hepatoma D23 cells. Applying these techniques, it has been established that during the early stages of subcutaneous growth of hepatoma D23 in syngeneic rats there is significant shedding of tumour specific antigen into the circulation. This is then depressed after a period of between 10 and 14 days tumour growth, at which time circulating tumour specific immune complexes are detectable. Finally, antibody can be detected. These observations are relevant to current views that cell mediated immunity to tumour cells can be blocked by interaction of antibody or immune complexes with tumour cells or by antigen inhibition of effector cells. Furthermore, these model studies indicate the importance of designing immunochemical assays for diagnostic and prognostic purposes during tumour therapy.